# A Transient Two−Phase Productivity Forecasting Method in Fractured Nanoporous Shale Gas Reservoirs

**DOI:** 10.3390/nano16040264

**Published:** 2026-02-17

**Authors:** Ruihan Zhang, Siliang He, Qianwen Zhang, Hongsha Xiao, Liehui Zhang

**Affiliations:** 1 State Key Laboratory of Oil & Gas Reservoir Geology and Exploitation, Southwest Petroleum University, Chengdu 610500, China; 2 Sichuan Changning Natural Gas Development Co., Ltd., Chengdu 610041, China

**Keywords:** shale gas, nanoporous media, multiphase flow, productivity forecasting, fracture interference

## Abstract

Hydraulic fracturing is a critical technology for developing shale gas reservoirs, which are typical natural nanoporous media. However, the complex two−phase flow induced by fracturing fluid retention and the strong interference among hydraulic fractures introduce significant uncertainties to productivity forecasting. To address these challenges, this study proposes a transient productivity forecasting method to characterize fluid transport in fractured nanoporous media. This method introduces a gas−water two−phase pseudo−pressure function to reconstruct the flow equations, utilizing micro−segment discretization and the principle of superposition to accurately characterize pressure drop interference among fractures, enabling rapid dynamic productivity forecasting under realistic well trajectory conditions. The investigation reveals that while increasing fracture count, half−length, and permeability enhances productivity, these improvements exhibit significant diminishing marginal returns, indicating the existence of optimal economic thresholds for these engineering parameters. Conversely, elevated water saturation, skin factor, and stress sensitivity lead to a decline in productivity. Analysis of flow interference demonstrates that fractures at the wellbore extremities contribute significantly higher production than those in the central section due to reduced interference, while deviations in the wellbore trajectory further exacerbate production heterogeneity. Field application confirms that the proposed method achieves reliable production history matching under realistic well trajectories and accurately captures the typical three−stage production characteristics of shale gas wells, providing a robust basis for Estimated Ultimate Recovery (EUR) assessment and fracturing design optimization.

## 1. Introduction

With the ongoing adjustment of the global energy structure, the strategic importance of shale gas has increased significantly. In 2024, shale gas production in the United States accounted for 79% of total natural gas production [1]. China also possesses abundant shale gas resources, particularly within the marine shale reservoirs of the Wufeng−Longmaxi Formation in the Sichuan Basin, which holds an estimated resource volume of 3.7 trillion cubic meters. From a material science perspective, the shale matrix is a natural nanoporous material [2,3,4,5]. Due to the complex nanoscale pore throats, shale reservoirs exhibit ultra−low permeability (typically below 0.0001 mD), necessitating hydraulic fracturing to establish complex fracture networks for economically feasible extraction. During this process, the two−phase flow induced by fracturing fluid retention and the strong interference among multiple fractures introduce significant uncertainties to productivity forecasting. Field data indicate that initial water production rates can reach up to 70%, with productivity typically declining by more than 60% within the first year [6,7,8]. Such transient production characteristics render traditional forecasting methods based on steady−state assumptions ineffective, often resulting in errors exceeding 35% [9]. This discrepancy leads to critical engineering challenges, including ineffective development plan optimization and inaccurate EUR assessments. Furthermore, in complex structural areas like the Sichuan Basin, well trajectory deviations exacerbate the heterogeneity of gas−water distribution [10]. Therefore, accurately forecasting the transient productivity of horizontal wells under realistic post−fracturing conditions has become a critical scientific issue that urgently needs to be addressed for efficient shale gas development.

Currently, shale gas productivity forecasting relies on four main approaches: decline curve analysis (DCA), analytical modeling, numerical simulation, and machine learning (ML). DCA offers computational simplicity by relying solely on historical production data, making it effective for early−stage evaluation. Various empirical models, including the Arps, power−law, extended exponential, and Duong methods, have been developed for diverse scenarios [11]. However, DCA lacks the capability to explicit account for geological and engineering parameters, leading to significant errors in long−term forecasting. In contrast, analytical models utilize simplified assumptions to derive mathematical solutions. While linear composite models [12] and type−curve integration methods [13] laid the groundwork, subsequent studies have advanced into dual−region models distinguishing the Stimulated Reservoir Volume (SRV) from the matrix [14], and multi−region matching techniques [15]. More recently, linear−radial composite models and models incorporating microscale mechanisms such as Knudsen diffusion have been proposed [16,17]. Advances have also been made in integrating fracture interference [18] and using source−sink theory for complex fracture systems [19]. Despite these developments, analytical models often sacrifice physical realism for efficiency, struggling to capture high heterogeneity and complex fracture geometries [20,21].

To address complex heterogeneities, numerical simulation and data−driven methods are widely adopted. Numerical approaches, such as the Discrete Fracture Network (DFN) [22] and the Embedded Discrete Fracture Model (EDFM) [23,24], precisely model coupled geomechanics and multiphase flow. Recent extensions have further enhanced EDFM for multiphase simulations [25] and coupled geological−fracture resolution [26]. Moreover, recent numerical studies have extensively investigated well interference mechanisms [27], complex fracture network evolution [28], and multi−layer hydraulic fracturing interactions [29]. However, the high computational cost and complex grid generation of these methods make them challenging for large−scale optimization or rapid decision−making. Conversely, ML methods, such as LSTM networks [30], weighted clustering algorithms [31], and multi−scale models incorporating fiber optic data [32], excel at capturing non−linear relationships. Yet, they often lack physical interpretability and generalization capability for new wells with distinct geological conditions.

Semi−analytical models incorporating numerical discretization have emerged as a balanced solution, bridging the gap between efficiency and accuracy. Yang et al. [33] established a transient model using point sink discretization, while He et al. [34] resolved two−phase flow complexities via a defined pseudo−pressure function. To better characterize reservoir heterogeneity and complex geometries, researchers have developed hybrid coupled models [35] and fractal induced fracture network models [36,37]. Additionally, models accounting for multi−scale seepage mechanisms [38], uncertainty quantification [39], and non−uniform SRV [40] have been proposed to enhance forecasting accuracy. Recent breakthroughs by Wu et al. [41,42] introduced efficient frameworks integrating stochastic fracture modeling and modified edge−based Green Element Methods (eGEM) to handle complex fracture networks and transient flow. Similarly, Cong et al. [43] developed models explicitly considering multi−well pressure interference. Despite these advancements, a unified framework that efficiently couples transient gas−water two−phase flow, multi−fracture interference, and realistic well trajectory effects remains elusive. Most existing models simplify fractures as uniform planes orthogonal to the wellbore, failing to capture the flow redistribution caused by actual trajectory deviations and the resulting non−uniform fracture interference.

To bridge these gaps, this study proposes a transient productivity forecasting method based on elastic transient flow theory. The distinct novelties of this work are threefold. First, unlike traditional semi−analytical models that simplify fractures as uniform planes orthogonal to the wellbore, it establishes a unified coupling framework that explicitly integrates realistic wellbore trajectories with complex fracture geometries, capturing the non−uniform flow interference often neglected by conventional approaches. Second, in contrast to models relying on single−phase or simplified multiphase assumptions, a gas−water two−phase pseudo−pressure function is derived to rigorously characterize the non−linear flow mechanisms in nanoporous media, specifically the hydraulic obstruction and Jamin effects induced by water retention. Third, distinguishing itself from computationally expensive numerical simulations, the model introduces a micro−segment discretization technique that achieves an optimal balance between physical fidelity and the computational efficiency required for rapid engineering decision−making. Furthermore, this study conducts a comprehensive sensitivity analysis to identify the diminishing marginal returns of engineering parameters and validates the model’s reliability through history matching and EUR forecasting.

## 2. Mathematical Model

### 2.1. Model Assumptions

**Figure 1:**
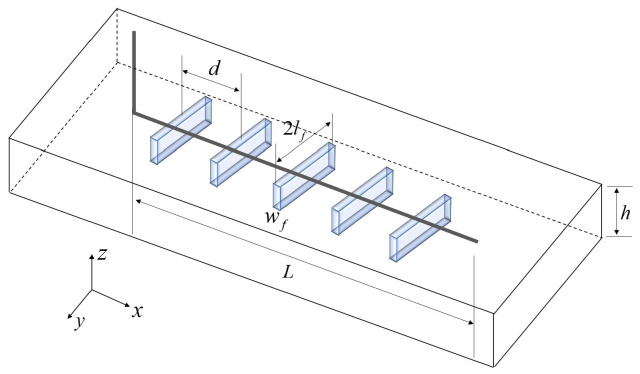
Multi−stage fracturing horizontal well in shale gas reservoir.

The physical model conceptualizes the multi−stage fractured horizontal well as an integrated hydraulic system, as illustrated in Figure 1. This system explicitly couples three distinct domains: the ultra−low permeability shale matrix, the hydraulic fracture network, and the deviated horizontal wellbore. To balance physical fidelity with computational efficiency for productivity forecasting and sensitivity analysis, the following fundamental assumptions are adopted:

1. The horizontal wellbore, with a length of L, is positioned at the geometric center of a reservoir assumed to be homogeneous and isotropic.
2. Hydraulic fracturing generates N primary fractures. The model explicitly accounts for geometric heterogeneity, including asymmetric fracture half−lengths, non−uniform fracture spacing, and arbitrary inclination angles relative to the wellbore axis;
3. Fluid flow follows a sequential path, solely from the shale matrix into the hydraulic fractures, and subsequently into the wellbore. Direct flow from the matrix to the wellbore is neglected due to the extreme permeability contrast.
4. The physical parameters of the hydraulic fractures are assumed to remain time−invariant during production to maintain analytical tractability.
5. The reservoir is treated as laterally extensive. During the transient flow period, the system behaves as an infinite−acting reservoir, with outer boundaries mathematically modeled at infinity.

### 2.2. Reservoir Flow and Point Source Solution

**Figure 2:**
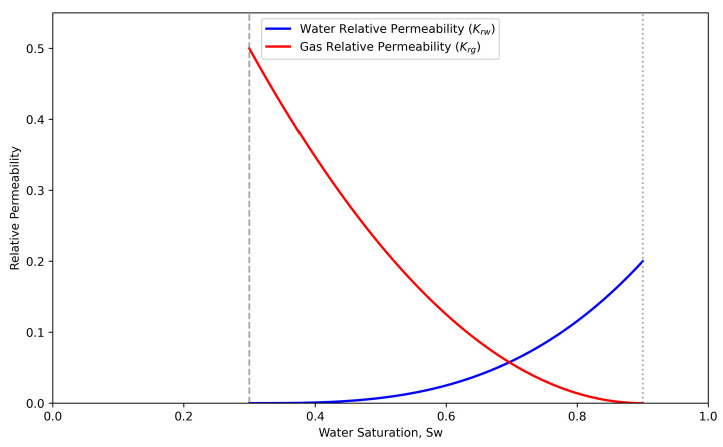
Relative permeability curves based on the Corey model.

Based on the theory of elastic transient flow, we analyze the fluid dynamics in an infinite, homogeneous, and isotropic reservoir. This theoretical framework is particularly suitable for shale gas reservoirs during the early and intermediate production stages. It effectively captures pressure propagation dominated by elastic rock−fluid behavior while simultaneously enabling the modeling of complex fracture interference via the superposition principle. The fundamental solution for the pressure drop caused by a continuous point sink is derived as follows [44]:

(1) $$p_{i}-p\left(x,y,t\right)=\frac{q\mu }{4\pi Kh}\left[-E_{i}\left(-\frac{{\left(x-x_{0}\right)}^{2}+{\left(y-y_{0}\right)}^{2}}{4\eta t}\right)\right]$$

where $p_{i}$ is the initial formation pressure; $p\left(x,y,t\right)$ is the pressure at location $\left(x,y\right)$ and time t; $\left(x_{0},y_{0}\right)$ denotes the point sink coordinates; q represents the gas production rate under standard conditions; K is the formation permeability; h is the reservoir thickness; μ is the effective viscosity; and η is the diffusivity coefficient. To characterize the non−linear two−phase flow dynamics in fractured horizontal wells, a pseudo−pressure function is formulated, defined as [45]:

(2) $$\psi \left(p\right)=\int_{0}^{p}\left(\frac{K_{rg}\rho_{g}\left(1+\delta /p\right)}{\mu_{g}}+\frac{K_{rw}\rho_{w}}{\mu_{w}}\right)e^{-\alpha \left(p_{i}-p\right)}dp$$

where $K_{rg}$ and $K_{rw}$ are the relative permeabilities of gas and water phases, respectively, defined using Corey−type correlations (as illustrated in Figure 2); $\rho_{g}$ and $\rho_{w}$ are phase densities; $\mu_{g}$ and $\mu_{w}$ are the phase viscosities; δ is the slippage factor; and α is the stress sensitivity coefficient.

**Figure 3:**
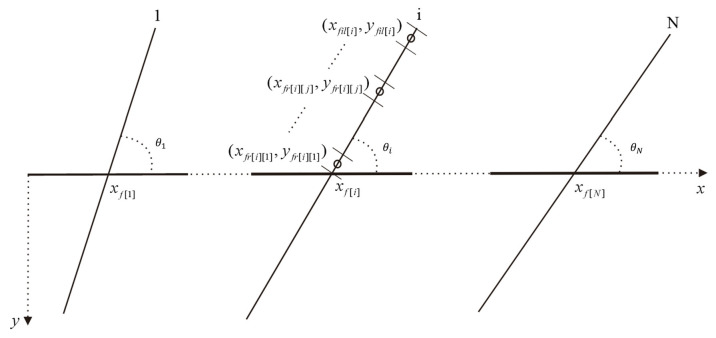
Fracture distribution along horizontal wells.

By combining the point sink solution with the pseudo−pressure definition, the governing equation for the potential drop at any reservoir location is obtained:

(3) $$\psi_{i}-\psi \left(x,y,t\right)=\frac{q\rho }{4\pi Kh}\left[-E_{i}\left(-\frac{{\left(x-x_{0}\right)}^{2}+{\left(y-y_{0}\right)}^{2}}{4\eta t}\right)\right]$$

where $\psi_{i}$ and $\psi \left(x,y,t\right)$ are the pseudo−pressures corresponding to pressure, and ρ is the effective density. To characterize the complex geometry of the hydraulic fracture network, the system is discretized into a series of continuous point sinks. Within this framework, the fracture−wellbore azimuth angle (denoted as θ) is explicitly incorporated by dividing each fracture wing into n equidistant micro−segments. Each micro−segment acts as a discrete point sink, with spatial coordinates determined via a coordinate transformation matrix relative to the wellbore axis. This geometric configuration for fractures with varying spatial orientations is illustrated in Figure 3.

Based on the principle of superposition, the total pressure field is decoupled into planar and vertical components. The planar pressure drop contribution from a specific fracture segment $\left(x_{fr\left[i\right]\left[j\right]},y_{fr\left[i\right]\left[j\right]}\right)$ is calculated as [45]:

(4) $$\Delta \psi_{Pr}=\frac{q_{fr\left[i\right]\left[j\right]}\rho }{4\pi Kh}\left[-E_{i}\left(-\frac{{\left(x-x_{fr\left[i\right]\left[j\right]}\right)}^{2}+{\left(y-y_{fr\left[i\right]\left[j\right]}\right)}^{2}}{4\eta t}\right)\right]$$

where $q_{fr\left[i\right]\left[j\right]}$ is the segment production rate of the j−th segment on the right wing of the i−th fracture, and the segment coordinates $\left(x_{fr\left[i\right]\left[j\right]},y_{fr\left[i\right]\left[j\right]}\right)$ are derived via the transformation equations [45]:

(5) $$\left\{\begin{array}{c} x_{fr\left[i\right]\left[j\right]}=x_{f\left[i\right]}+\frac{2j-1}{2n}\cdot l_{fr\left[i\right]}\cdot \cos\left(\theta_{i}\right)\\ y_{fr\left[i\right]\left[j\right]}=y_{f\left[i\right]}+\frac{2j-1}{2n}\cdot l_{fr\left[i\right]}\cdot \sin\left(\theta_{i}\right) \end{array}\right.$$

where $l_{fr\left[i\right]}$ is the length of the right fracture wing; n represents the number of discrete micro−segments per wing; and $\theta_{i}$ is the azimuth angle between the fracture plane and the horizontal wellbore axis. To account for elevation differences induced by wellbore trajectory deviation and fracture dip, a gravitational potential correction term is introduced [46]:

(6) $$\Delta \psi_{Gr}=\frac{\rho^{2}g\left(z-z_{fr\left[i\right]\left[j\right]}\right)}{\mu }-\frac{\rho^{2}g\left(z_{ref}-z_{fr\left[i\right]\left[j\right]}\right)}{\mu }$$

where z denotes the vertical coordinate of the observation point; $z_{ref}$ represents the reference elevation datum; $z_{fr\left[i\right]\left[j\right]}$ is the vertical coordinate of the j−th micro−segment on the right wing of the i−th fracture; and g is the gravitational acceleration. The gravitational term is introduced to explicitly account for the vertical potential difference caused by elevation changes, particularly in scenarios involving inclined wellbores or spatially complex fracture networks. Consequently, the cumulative pseudo−pressure drop at any spatial coordinate $\left(x,y,z\right)$ and time t, induced by the right wing of fracture i, is determined by the summation of individual sink contributions:

(7) $$\psi_{i}-\psi \left(x,y,z,t\right){|}_{r,i}=\sum_{j=1}^{n}\left[\Delta \psi_{Pr}+\Delta \psi_{Gr}\right]$$

A corresponding computational procedure is applied to the left fracture wing. Consequently, for a reservoir system containing N hydraulic fractures, the cumulative pseudo−pressure drop at any spatial location $\left(x,y,z\right)$ and time t is derived via the linear superposition of contributions from all individual fracture wings:

(8) $$\psi_{i}-\psi \left(x,y,z,t\right){|}_{N}=\sum_{i=1}^{n}\left[{(\psi }_{i}-\psi \left(x,y,z,t\right){|}_{r,i})+{(\psi }_{i}-\psi \left(x,y,z,t\right){|}_{l,i})\right]$$

### 2.3. Matrix−Fracture Coupled Flow

The coordinate of the right−wing tip of the i−th fracture is $\left(x_{fir\left[i\right]},y_{fir\left[i\right]},z_{fir\left[i\right]}\right)$. According to Equation (7), the cumulative pseudo−pressure drop at this specific location, induced by the interference of the entire fracture network at time t, is formulated as:

(9) ψi−ψfiri=∑k=1N∑j=1nqfrkjρ4πKh−Ei−xfiri−xfrkj2+yfiri−yfrkj24ηt+ΔψGr+∑j=1nqflkjBρ4πKh−Ei−xfiri−xflkj2+yfiri−yflkj24ηt+ΔψGl

where $\psi_{fir\left[i\right]}$ represents the pseudo−pressure at the right−wing tip of fracture i. To account for asymmetric fracture wing lengths, the effective pseudo−pressure of fracture is approximated by the arithmetic mean of the potentials at both wing tips [47]:

(10) $$\psi_{fi\left[i\right]}=\frac{\psi_{fir\left[i\right]}+\psi_{fil\left[i\right]}}{2}$$

### 2.4. Fracture−Wellbore Coupled Flow and System Solution

**Figure 4:**
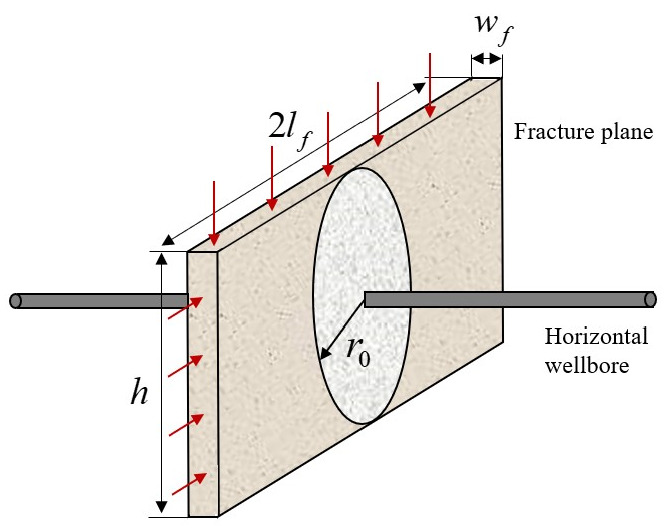
Schematic of the fracture−wellbore flow.

The hydraulic fracture functions as a finite−conductivity conduit connecting the shale matrix to the horizontal wellbore. The flow dynamics within this domain are characterized by the convergence of fluid from the fracture network into the wellbore. The outer boundary condition is defined by the effective pseudo−pressure at the fracture tip, $\psi_{fi\left[i\right]}$, while the inner boundary condition is governed by the flowing bottom−hole pseudo−pressure at the wellbore, $\psi_{wf\left[i\right]}$. The linear−to−radial flow and its associated geometric parameters are shown in Figure 4.

The pseudo−pressure drop from the fracture tip to the wellbore is mathematically modeled using a modified radial flow equation [45]:

(11) $$\psi_{fi\left[i\right]}-\psi_{wf\left[i\right]}=\frac{q_{f\left[i\right]}\rho }{2\pi K_{f\left[i\right]}w_{f\left[i\right]}}\left(\frac{\ln\sqrt{\left(l_{fr\left[i\right]}+l_{fl\left[i\right]}\right)h/\pi }}{r_{w}}+s\right)$$

where $q_{f\left[i\right]}$ represents the total production rate of the i−th fracture; $K_{f\left[i\right]}$ and $w_{f\left[i\right]}$ denote the fracture permeability and width, respectively; $r_{w}$ is the radius of the horizontal wellbore; and s is the total skin factor. This fracture−wellbore coupling model is adapted from classical approaches [48,49,50], but extends previous works by integrating the non−linear two−phase pseudo−pressure function and the micro−segment discretization framework. To bridge the macro−scale fracture production ($q_{f\left[i\right]}$) with the micro−scale point sinks, the flow rate is allocated to the j−th segment of the k−th fracture using a geometric weighting approach:

(12) $$\left\{\begin{array}{c} q_{fr\left[k\right]\left[j\right]}=\frac{1}{n}\frac{l_{fr\left[k\right]}}{l_{fr\left[k\right]}+l_{fl\left[k\right]}}q_{f\left[k\right]}\\ q_{fl\left[k\right]\left[j\right]}=\frac{1}{n}\frac{l_{fl\left[k\right]}}{l_{fr\left[k\right]}+l_{fl\left[k\right]}}q_{f\left[k\right]} \end{array}\right.$$

This allocation strategy assumes a uniform flux distribution proportional to the wing length. While this simplifies the complex intra−fracture pressure gradients, it provides a computationally efficient approximation for productivity forecasting.

Adopting the infinite−conductivity wellbore assumption (negligible frictional pressure drop), the pseudo−pressure at each fracture−wellbore intersection is considered uniform and identical to the bottom−hole flowing pseudo−pressure ($\psi_{wf}$). By strictly coupling the Matrix−Fracture flow solution with the Fracture−Wellbore flow equation and substituting the production allocation scheme, the composite pseudo−pressure drop for the entire system is synthesized as:

(13) $$\psi_{i}-\psi_{wf}=\left(\psi_{i}-\psi_{fi\left[i\right]}\right)+\left(\psi_{fi\left[i\right]}-\psi_{wf}\right)$$

Finally, the total well production rate is given by the linear superposition of the entire hydraulic fracture network [51]:

(14) $$q=\sum_{i=1}^{N}q_{f\left[i\right]}$$

## 3. Results

### 3.1. Sensitivity Analysis of Key Parameters

A systematic sensitivity analysis is conducted using an idealized base case model characterized by uniformly spaced fractures and homogeneous reservoir properties. The key input parameters are summarized in Table 1.

#### 3.1.1. Impact of Fracture Number

**Figure 5:**
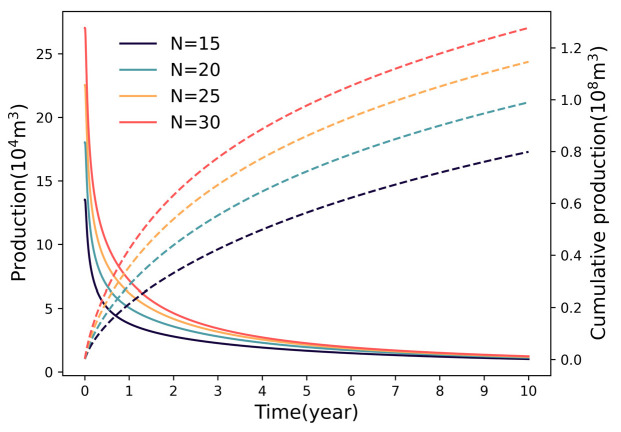
Variation in productivity with different fracture numbers.

Figure 5 illustrates the production performance under varying fracture densities. As the number of fractures increases from 15 to 30, the cumulative gas production rises from 0.79 × 10^8^ m^3^ to 1.28 × 10^8^ m^3^. However, this growth follows a distinct trend of diminishing marginal returns, with the incremental gain diminishing significantly (25.31% → 16.16% → 12.17%). This trend reflects the competitive mechanism within the multi−fracture system: while a higher fracture density expands the total SRV, it simultaneously reduces the inter−fracture spacing. Consequently, severe interference occurs between adjacent fractures, diminishing the effective drainage area and contribution of individual fractures.

#### 3.1.2. Impact of Fracture Half−Length

**Figure 6:**
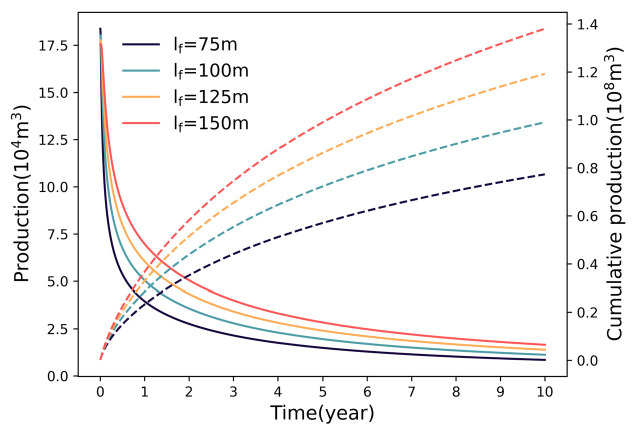
Variation in productivity with different fracture half−lengths.

The sensitivity of cumulative production to fracture half−length is depicted in Figure 6. Extending the half−length from 75 m to 150 m boosts production from 0.77 × 10^8^ m^3^ to 1.38 × 10^8^ m^3^, yet the enhancement rate shows a downward trajectory (28.57% → 20.21% → 15.96%). This non−linear response highlights the trade−off between drainage volume and flow efficiency. Although longer fractures penetrate deeper into the matrix, the pressure gradient along the fracture length degrades at distal ends. When the driving pressure differential drops below a critical threshold, the flow contribution from the fracture tips becomes negligible, limiting the efficacy of further length extension.

#### 3.1.3. Impact of Fracture Permeability

**Figure 7:**
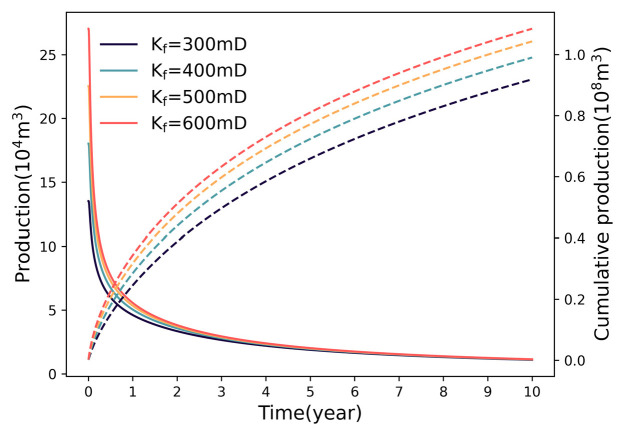
Variation in productivity with different fracture permeabilities.

Figure 7 demonstrates the dynamic influence of fracture conductivity. Increasing fracture permeability from 300 mD to 600 mD yields a moderate production rise from 0.91 × 10^8^ m^3^ to 1.08 × 10^8^ m^3^. Notably, the magnitude of this increase diminishes rapidly (8.79% → 5.05% → 3.84%). This phenomenon indicates that once fracture permeability exceeds a certain threshold, the flow bottleneck shifts from the fracture conduit to the ultra−low permeability matrix. Consequently, further increasing fracture conductivity provides minimal benefit to late−stage productivity.

#### 3.1.4. Impact of Fracture Orientation

**Figure 8:**
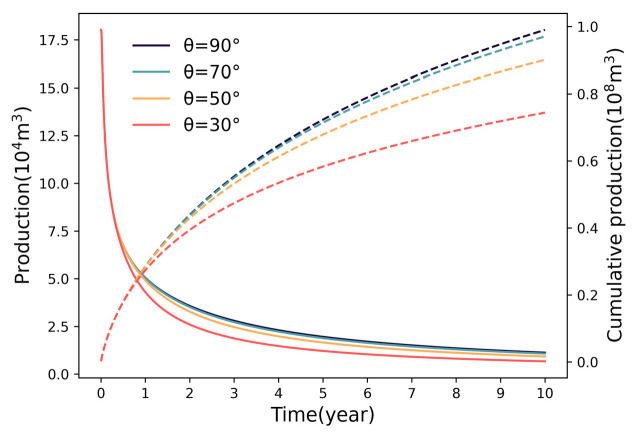
Variation in productivity with different fracture angles.

The geometric relationship between the fracture and wellbore plays a critical role in flow efficiency. As shown in Figure 8, adjusting the fracture−wellbore angle (θ) from 30° to 90° increases production from 0.74 × 10^8^ m^3^ to 0.99 × 10^8^ m^3^. The declining growth rate (22.97% → 8.79% → 2.02%) suggests that the penalty for mild deviation is manageable, but severe inclination is detrimental. Theoretically, oblique fractures increase the tortuosity and length of the flow path from the matrix to the wellbore, thereby escalating flow resistance. Therefore, maintaining hydraulic fractures strictly perpendicular to the horizontal wellbore is essential for minimizing pressure drops.

#### 3.1.5. Impact of Water Saturation

**Figure 9:**
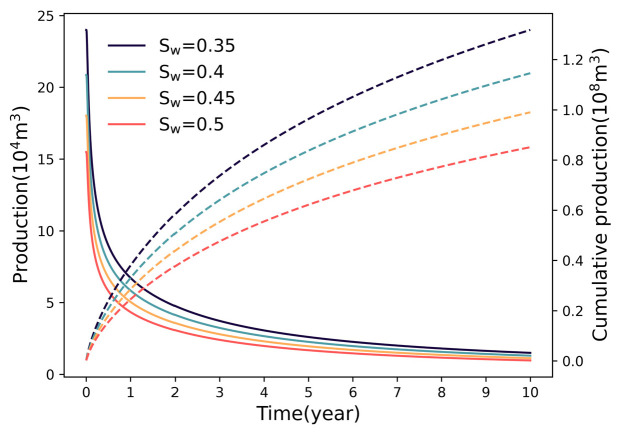
Variation in productivity with different water saturation.

Water saturation ($S_{w}$) exerts a strong negative control on gas productivity. As illustrated in Figure 9, increasing $S_{w}$ from 0.35 to 0.50 causes a sharp decline in cumulative production from 1.32 × 10^8^ m^3^ to 0.85 × 10^8^ m^3^. The accelerating decline rate (reaching 14.14%) underscores the non−linear nature of the relative permeability curves. Beyond a critical saturation ($S_{w}$ > 0.45), water preferentially occupies the larger pore throats and micro−fractures due to capillary forces. This occupation restricts the effective flow pathways for the gas phase, significantly impairing gas mobility. Furthermore, in nanoporous shale media, the interaction between gas and water induces severe Jamin effects, where liquid droplets trapped in pore throats create additional capillary resistance. This phenomenon leads to an exponential increase in gas flow resistance, as reflected by the steep decline in the gas relative permeability curve (Figure 2). Consequently, the pseudo−pressure drop required to drive gas flow increases disproportionately, resulting in the observed sharp decline in well productivity.

#### 3.1.6. Impact of Skin Factor

**Figure 10:**
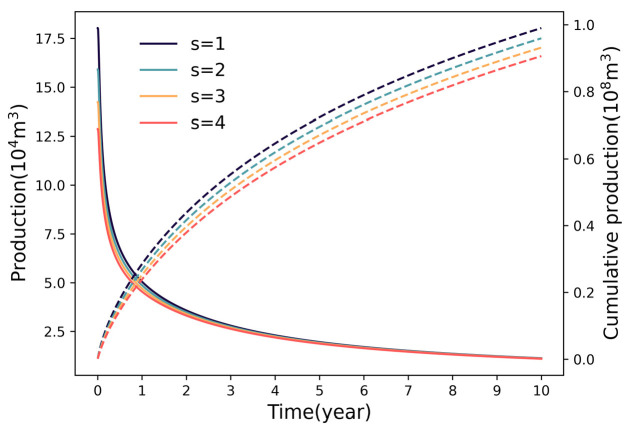
Variation in productivity with different skin factors.

Figure 10 reveals a linear negative correlation between the skin factor and well performance. As the skin factor rises from 1 to 4, cumulative gas production drops from 0.99 × 10^8^ m^3^ to 0.91 × 10^8^ m^3^, averaging a 2.7% loss per unit increase. Analysis indicates that an increase in the skin factor leads to elevated flow resistance near the wellbore. It is recommended to control the skin factor below 2 through composite acid stimulation and optimization of proppant concentration to mitigate productivity loss.

### 3.2. Case Study

**Figure 11:**
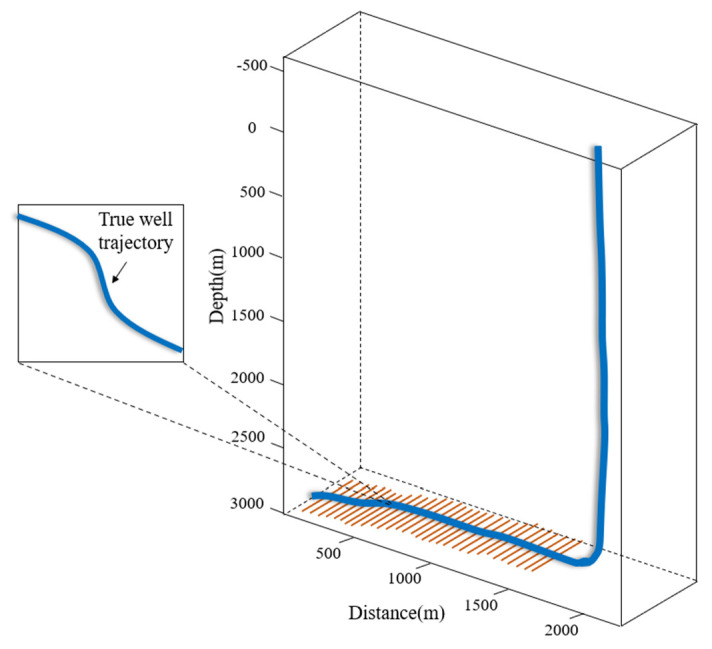
Schematic of the well trajectory and fracture distribution.

To validate the practical utility and accuracy of the proposed model, a comprehensive case study was conducted on Well S−1, a representative multi−stage fractured horizontal well located in the Longmaxi Formation of the southern Sichuan Basin. The reservoir matrix exhibits ultra−low permeability and developed natural micro−fractures, necessitating massive hydraulic stimulation. The spatial configuration of the wellbore and hydraulic fractures is illustrated in Figure 11. To facilitate a clear understanding of the simulation background, the key geological characteristics and engineering parameters of Well S−1 are summarized in Table 2.

#### 3.2.1. History Matching

**Figure 12:**
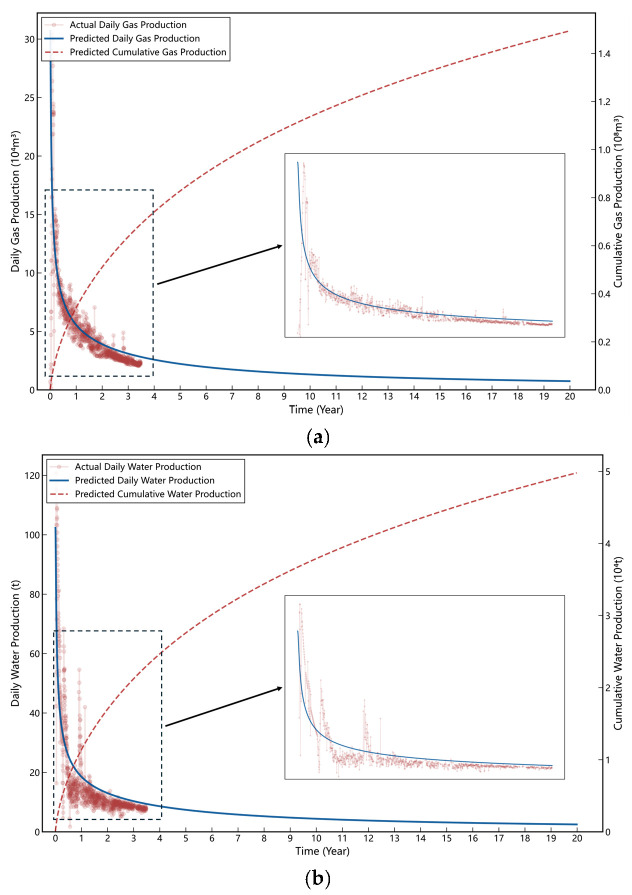
Comparison of observed and simulated production history for Well S−1: (**a**) Gas production; (**b**) Water production.

To validate the accuracy and reliability of the proposed model, a rigorous production history matching study was performed using the first four years of field data from Well S−1. Figure 12 presents the comparison between the simulated production dynamics and the actual historical production records.

The simulated curves exhibit a high degree of consistency with the field observations. The model accurately captures the characteristic flow regimes of the shale gas well, specifically reproducing the rapid decline in the early stage and the gradual stabilization in the late stage. To strictly quantify the matching quality, statistical performance metrics are summarized in Table 3.

The validation results confirm the model’s strong predictive capabilities, particularly for the gas phase. The gas production simulation achieves a high coefficient of determination (R^2^ = 0.7933) combined with a low MAPE (17.54%), demonstrating a rigorous agreement between simulated and observed values. Regarding water production, the model exhibits acceptable predictive capability with an R^2^ of 0.7244. While the higher MAPE reflects the inherent variability associated with the low−magnitude and erratic nature of field water rates, the model successfully captures the overall water−cut trend. These metrics establish the utility of the proposed model for accurate transient productivity forecasting in multi−stage fractured horizontal wells.

#### 3.2.2. Analysis of Fracture Productivity Heterogeneity

**Figure 13:**
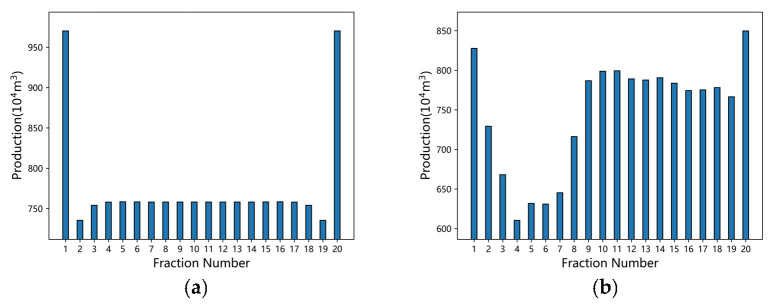
Fracture productivity distribution: (**a**) Idealized model; (**b**) Actual distribution for Well S−1.

An advantage of the proposed model is its capability to resolve the contribution of fractures, providing critical insights for hydraulic fracturing optimization. Figure 13 illustrates the distribution of cumulative gas production for each fracture stage after a 20−year production period, comparing an idealized scenario with the actual field case (Well S−1).

In the idealized mechanism model, the inter−fracture interference results in a characteristic distribution where the “heel” and “toe” fractures exhibit significantly higher productivity than the central ones. Specifically, the exterior fractures reach a cumulative production of 530 × 10^4^ m^3^, while the interior fractures remain relatively uniform, with a production of approximately 490 × 10^4^ m^3^. In the actual well trajectory scenario, the central fractures display a much higher degree of heterogeneity. Notably, the production of certain fractures drops precipitously to as low as 380 × 10^4^ m^3^. This pronounced non−uniformity is attributed to the combined effects of irregular fracture spacing and the undulating nature of the realistic wellbore trajectory.

## 4. Discussion

### 4.1. Diminishing Marginal Returns of Fracture Parameters

The multi−parameter sensitivity analysis in this study reveals a consistent trend of diminishing marginal returns regarding fracture geometry (number, half−length) and physical properties (permeability). While intensifying these parameters theoretically expands the contact area with the matrix, the production gain does not scale linearly. This phenomenon is fundamentally governed by the competitive interaction between SRV expansion and inter−fracture pressure interference. Specifically, increasing fracture density creates a trade−off: it multiplies flow pathways but simultaneously compresses the inter−fracture spacing. As the spacing decreases, the pressure depletion zones of adjacent fractures overlap significantly, intensifying the interference field and reducing the effective drainage volume of individual fractures. Similarly, for fracture half−length, the driving pressure gradient at the distal tips degrades as length increases, rendering the extended segments less effective in contributing to flow. Regarding permeability, once it exceeds a certain threshold, the flow bottleneck shifts from the fracture conduit to the ultra−low permeability matrix, making further conductivity enhancement redundant.

Consequently, these findings suggest the existence of an “optimal range” for fracture parameters. Within this range, the expansion of the drainage network effectively dominates over interference effects. However, beyond this critical threshold, the incremental productivity gains become marginal and fail to justify the escalating engineering costs. Therefore, fracture design should not solely pursue maximization but rather aim to identify these economic inflection points to balance reservoir stimulation efficiency with capital investment.

### 4.2. Mechanisms of Fracture Interference and Multiphase Flow

The transient productivity behavior of fractured shale gas wells is governed by a complex interplay of mechanisms spanning from the macro−scale fracture network to the micro−scale nanopores.

At the macro−scale, the phenomenon of diminishing marginal returns observed in fracture densification is fundamentally driven by the superposition of pressure transient waves. As defined by Equation (8), the potential drop at any point is the cumulative sum of contributions from all discrete fracture segments. When fracture spacing decreases, the drainage areas of adjacent fractures overlap extensively. This overlap reduces the effective pressure gradient driving fluid from the matrix into the fracture face. Consequently, while the total SRV increases, the flux contribution per unit fracture surface area degrades. This mechanism explains the significant heterogeneity observed in the field case (Figure 13), where central fractures, subjected to interference from both sides, exhibit lower productivity compared to the “heel” and “toe” fractures which benefit from semi−unbounded drainage boundaries.

The micro−scale flow efficiency is dominated by the competitive flow between gas and water phases, particularly during the early load−up and flowback stages. Hydraulic fracturing injects massive volumes of fluid, creating a high−water−saturation zone near the fracture face. Physically, this retained water occupies the larger pore throats and primary flow channels, significantly reducing the effective cross−sectional area available for gas transport. In our model, this phenomenon is quantified through the relative permeability curves (Figure 2), where a rise in water saturation induces a sharp penalty on gas relative permeability ($K_{rg}$). This accumulation of water forms a hydraulic obstruction that significantly restricts gas from flowing from the matrix into the hydraulic fractures, which explains the steep production decline often observed in the initial production period. Furthermore, the unique nanoporous structure of shale reservoirs introduces severe capillary−induced resistance, known as the Jamin effect [52,53]. Beyond simple volume occupation, liquid droplets trapped in nanoscale pore throats require a significantly higher pressure differential to be displaced. As the gas phase attempts to bypass these water−filled throats, the additional capillary resistance manifests as a non−linear increase in apparent viscosity and a reduction in effective permeability. This mechanism is particularly dominant in ultra−low permeability zones where pore throat sizes are comparable to the mean free path of gas molecules. By integrating these effects into the pseudo−pressure integral, the model accurately reflects why productivity is so sensitive to water saturation changes.

### 4.3. Model Limitations

While the proposed semi−analytical model achieves a balance between computational efficiency and physical fidelity for transient productivity forecasting, it is subject to certain limitations imposed by its underlying assumptions:

1. The model assumes a homogeneous reservoir to ensure analytical tractability. Consequently, in formations with significant heterogeneity (e.g., layered structures, anisotropic stress, or strong permeability contrasts), the model may underrepresent localized preferential flow pathways, leading to potential deviations in pressure predictions.
2. The flow pattern is conceptualized as a primary fracture system. The model neglects complex natural fracture networks and secondary branch fractures. This omission may result in an oversimplified representation of fracture networks.
3. To balance efficiency and accuracy, this study adopts an effective continuum approach. While the proposed pseudo−pressure function implicitly integrates nanoconfinement effects (such as gas slippage), mechanisms like adsorption and diffusion are not explicitly formulated. Future work will focus on upscaling these mechanisms into the model to further enhance prediction accuracy.

## 5. Conclusions

In this study, a novel semi−analytical productivity forecasting method for multi−stage fractured horizontal wells in shale gas reservoirs is proposed. By integrating a gas−water two−phase pseudo−pressure function with a micro−segment discretization technique, the model explicitly couples transient two−phase flow, complex fracture interference, and realistic wellbore trajectories within a unified framework. Based on the sensitivity analysis and field case validation, the following conclusions are drawn:

1. The proposed method demonstrates high accuracy in reproducing transient production history. Validated against field data from Well S−1, the model achieved a high coefficient of determination for gas production (R^2^ = 0.7933) and captured the general trend of water production. This confirms its capability to serve as an effective tool for EUR assessment and dynamic performance analysis.
2. Sensitivity analysis elucidates that while increasing fracture number, half−length, and permeability enhances cumulative production, the incremental gains exhibit significant diminishing marginal returns. This phenomenon is governed by the competitive interaction between the expansion of the SRV and the intensification of inter−fracture pressure interference. Consequently, unconstrained maximization of fracture parameters is inefficient. Instead, optimal economic thresholds should be identified to balance production gains with capital investment.
3. Research indicates that fracture productivity exhibits significant heterogeneity driven by fracture interference. In idealized scenarios, external fractures exhibit higher productivity than central ones, benefiting from reduced interference intensity. However, under realistic field conditions, wellbore trajectory deviations further increase this heterogeneity, resulting in pronounced productivity disparities among fractures. This underscores the critical importance of incorporating actual wellbore trajectories into productivity forecasting to ensure predictive accuracy.

The present framework assumes a homogeneous reservoir and primary planar fractures to maintain analytical tractability, which may oversimplify the complexities of naturally fractured formations. Therefore, future work will focus on extending this semi−analytical approach to incorporate Discrete Fracture Networks (DFN) and explicitly couple geomechanical effects. Additionally, upscaling micro−scale mechanisms, such as adsorption and Knudsen diffusion, remains a key objective to further enhance prediction accuracy for deep shale gas reservoirs.

## Figures and Tables

**Table 1:** Parameters of the base mechanism model.

Parameter	Value	Parameter	Value
Reservoir thickness	100 m	Matrix permeability	0.0001 mD
Matrix porosity	0.03	Total skin factor	1
Horizontal well length	1000 m	Wellbore radius	0.12 m
Initial reservoir pressure	35 MPa	Bottom−hole pressure	20 MPa
Fracture width	0.005 m	Number of fractures	20
Fracture half−length	100 m	Fracture permeability	400 mD
Production duration	10 years	Water saturation	0.35

**Table 2:** Reservoir and fracture parameters of the case study well (Well S−1).

Parameter	Value	Parameter	Value
Reservoir thickness	87 m	Matrix permeability	0.00021 mD
Matrix porosity	0.052	Water saturation	0.22
Horizontal well length	1800 m	Wellbore radius	0.108 m
Initial reservoir pressure	58 MPa	Bottom−hole pressure	28 MPa
Number of fractures	27	Fracture width	0.005 m
Fracture half−length	95 m	Fracture permeability	400 mD

**Table 3:** Error analysis and matching accuracy metrics for history matching.

	MAE	RMSE	MAPE	R^2^
Gas	8929.8	18,412.15	17.54%	0.7933
Water	5.9	9.59	35.64%	0.7244

## Data Availability

The data are contained within this article.
